# TIMP-1 activated carcinoma-associated fibroblasts inhibit tumor apoptosis by activating SDF1/CXCR4 signaling in hepatocellular carcinoma

**DOI:** 10.18632/oncotarget.3616

**Published:** 2015-04-01

**Authors:** Tao Song, Changwei Dou, Yuli Jia, Kangsheng Tu, Xin Zheng

**Affiliations:** ^1^ Department of Hepatobiliary Surgery, the First Affiliated Hospital of Xi'an Jiaotong University, Xi'an, Shaanxi 710061, China

**Keywords:** TIMP-1, CAFs, HCC, apoptosis, tumor microenvironment

## Abstract

Tissue inhibitor of metalloproteinase 1 (TIMP-1) is an endogenous inhibitor for MMPs that regulates the remodeling and turnover of the ECM during normal development and pathological conditions. Intriguingly, recent studies have shown that TIMP-1 plays a dual role in cancer progression. In this study, we found that TIMP-1 expression in HCC tissues is associated with advanced TNM stage, intrahepatic metastasis, portal vein invasion, and vasculature invasion. Notably, TIMP-1 expression in HCC tissue is significantly related to worse overall survival for patients with HCC after liver resection. Ectopic TIMP1 expression promoted the growth of HCC xenografts in nude mice. Both co-culture with Huh7 cells with a high level of TIMP-1 and TIMP1 treatment resulted in up-regulation of hallmarks of carcinoma-associated fibroblasts (CAFs) and accelerated cell proliferation, migration and invasion in immortalized liver fibroblasts (LFs) isolated from human normal liver tissue. By co-culture with CAFs, SDF-1/CXCR4/PI3K/AKT signaling was activated and apoptosis was markedly repressed with an increased Bcl-2/BAX ratio in Huh7 cells. Taken together, our observations suggest that TIMP-1 induces the trans-differentiation of LFs into CAFs, suppresses apoptosis via SDF-1/CXCR4/PI3K/AKT signaling and then promotes HCC progression. This protein may be a potential prognostic biomarker and therapeutic target for HCC.

## INTRODUCTION

Hepatocellular carcinoma (HCC) is an aggressive cancer that frequently occurs in the setting of chronic hepatitis infection and liver cirrhosis. This cancer has been identified as the fifth most common cancer and third leading cause of cancer-related deaths worldwide [1]. Liver resection has been considered to be the main curative therapy for HCC worldwide over a long period of time. Despite advances in therapeutic modalities during the past decade, the prognosis of HCC after liver resection remains dismal due to the high rate of recurrence and metastasis induced by yet unknown biological factors. Hence, it is critical to explore the molecular mechanisms controlling HCC recurrence and metastasis to develop new therapeutic strategies for this disease.

Cancer progression is a highly complicated process during which a variety of cells, including malignant and stromal cells, interact with each other in the cancer microenvironment. The extracellular matrix (ECM), consisting of structural proteins such as collagen, elastin, fibronectin, and laminin, provides a structural framework for the cancer microenvironment and functions as a barrier or promoting environment [2, 3]. Matrix metalloproteinases (MMPs) are zinc-dependent proteolytic enzymes involved in the remodeling of the extracellular matrix by breaking down basement membranes and most extracellular matrix (ECM) components [4]. It has been reported that the activity of MMPs may be modulated by proenzyme activation of endogenous natural inhibitors i.e., tissue inhibitor of matrix metalloproteinases (TIMPs) [5, 6]. TIMP-1 is a member of the TIMP family and inhibits MMP proteolytic activity by forming noncovalent 1:1 stoichiometric complexes that are resistant to heat denaturation and proteolytic degradation [7]. Different studies have shown that TIMP-1 has both cell proliferation and anti-oncogenic effects. Overexpression of TIMP-1 was found in pancreatic cancer [8], laryngeal squamous cell carcinoma [9] and lung adenocarcinoma [10], whereas its expression is lost in prostate adenocarcinoma [11]. It appears that TIMP-1 exerts different effects on tumor progression in various cancers. To date, there are limited studies of the role of TIMP-1 in HCC progression.

Cancer tumors are complex organs composed not only of original neoplastic cells but also carcinoma-associated stromal cells and an extracellular milieu including various chemokines and cytokines. Cancer progression partly results from an evolving interaction between founder neoplastic cells and their surrounding carcinoma-associated stromal cells and supportive tissue. Carcinoma-associated fibroblasts (CAFs) are critical components of carcinoma-associated stromal cells, which are characterized by the overexpression of α-smooth muscle actin (α-SMA), fibroblast activation protein (FAP), fibroblast surface protein (FSP), and vimentin. CAFs have been extracted from HCC [12] and proven to promote HCC progression. However, the underlying mechanism of these cells remains largely unknown. Furthermore, it is unclear how normal liver fibroblasts (LFs) are transformed into CAFs in the HCC microenvironment.

In this study, we detected TIMP-1 expression in HCC samples and elucidated its role in the transdifferentiation of LFs into CAFs.

## RESULTS

### TIMP-1 is up-regulated in HCC and predicts poor post-surgical survival

**Figure 1:**
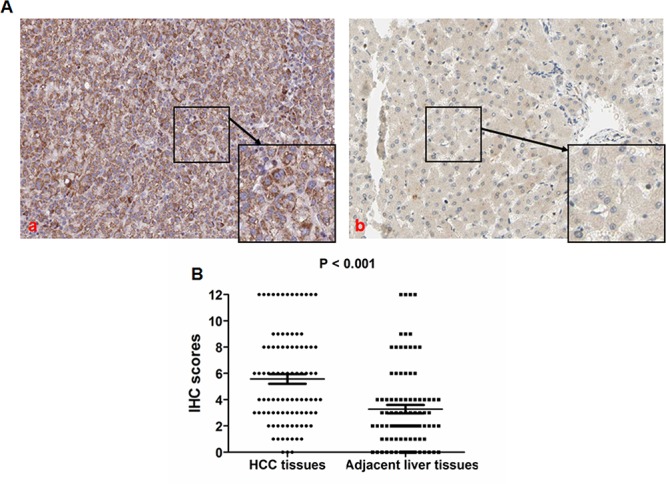
TIMP-1 expression is up-regulated in HCC tissues **A.** TIMP-1 protein is mainly expressed in the cytoplasm of tumor cells, and TIMP-1 expression in HCC tissues was remarkably higher (a) compared with adjacent liver tissues (b). **B.** As shown in the vertical scatter plot, the IHC scores in the TIMP-1 high group (mean value: 5.57) was notably higher than that in the TIMP-1 low/non group with a mean value 3.28 (*P* < 0.001) after analysis by the Mann-Whitney *U* test.

The results of IHC revealed the cytoplasmic staining of the TIMP-1 protein (Figure 1A). Positive TIMP-1 protein expression was observed in 94/100 (94%) HCC cases. TIMP-1 expression was increased in tumor cells compared with benign tissues in 76.6% of the HCC tumors examined. As assessed by the Mann-Whitney *U* test, it was demonstrated that TIMP-1 expression is significantly higher in HCC tissues compared with adjacent liver tissues (*P* < 0.001, Figure 1B). The relationship between TIMP-1 and the clinicopathological parameters of 100 HCCs was statistically examined, and the results are listed in Table 1. TIMP-1 expression in HCC tissues was remarkably related to Edmonson–Steiner classification (*r* = 8.16, *P* = 0.004), tumor node metastasis (TNM) stage (*r* = 8.39, *P* = 0.004), portal vein invasion (*r* = 11.94, *P* < 0.001) and intrahepatic metastases (*r* = 13.09, *P* < 0.001), whereas no significant correlation was found between TIMP-1 expression in HCC tissues and gender (*r* = 0.21, *P* = 0.647), age (*r* = 2.89, *P* = 0.089), HBV infection (*r* = 0.31, *P* = 0.578), liver cirrhosis (*r* < 0.01, *P* = 0.955), serumα-fetoprotein (AFP) level (*r* = 0.79, *P* = 0.374), tumor size (*r* = 2.42, *P* = 0.120), and vasculature invasion (*r* = 0.39, *P* = 0.533).

**Table 1:** Relationship between clinicopathological features and TIMP-1 expression in tumor tissues from 100 HCC patients

Clinicopathological features		No. of patients		χ^2^	*p*
		TIMP-1 high	TIMP-1 low/non		
Age (year)	< 50	24	8	0.21	0.647
	≥ 50	48	20		
Gender	Male	53	25	2.89	0.089
	Female	19	3		
HBV infection	Present	60	22	0.31	0.578
	Absent	12	6		
Serum AFP level (ng/mL)	≥ 400	53	34	0.79	0.374
	< 400	19	8		
Tumor size (cm)	≥ 5	53	25	2.42	0.120
	< 5	19	3		
Liver cirrhosis	Present	64	25	< 0.01	0.955
	Absent	8	3		
Vasculature invasion	Present	26	12	0.39	0.533
	Absent	46	16		
Edmondson-Steiner Classification	I + II	14	13	8.16	0.004
	III + IV	58	14		
TNM stage	I + II	46	26	8.39	0.004
	III + IV	26	2		
Portal vein invasion	Present	7	11	11.94	< 0.001
	Absent	65	17		
Intrahepatic metastases	Present	5	10	13.09	< 0.001
	Absent	67	18		

**Figure 2:**
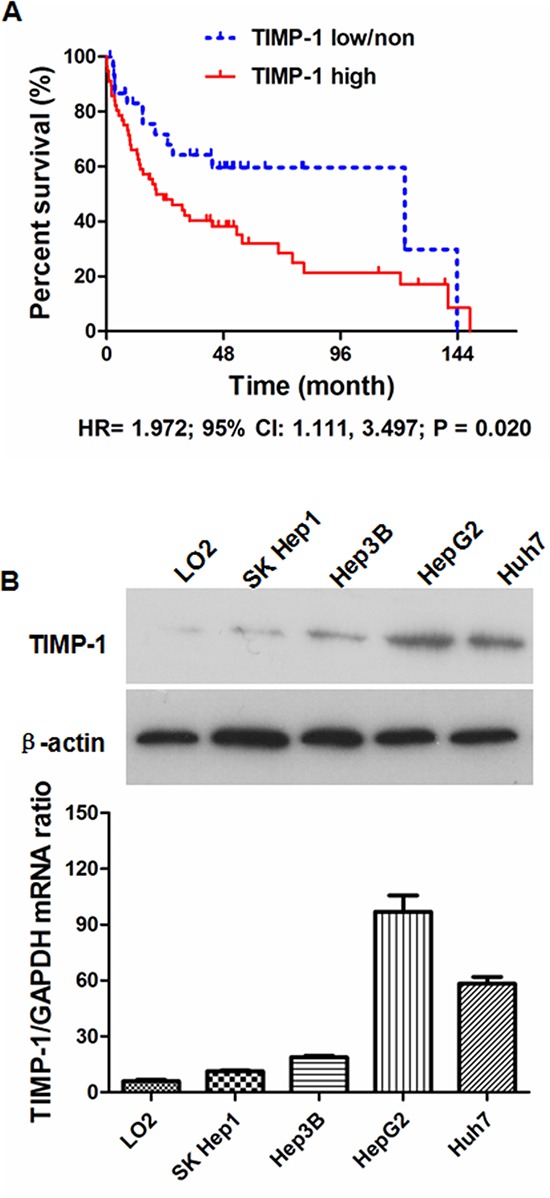
Aberrant overexpression of TIMP-1 in HCC tissues was associated with worse outcome after liver resection **A.** Kaplan-Meier survival curves demonstrate that the TIMP-1 high group have a shortened post-surgical survival time compared with the TIMP-1 low/non group (*HR* = 1.972; 95% CI: 1.111, 3.497; *P* = 0.020). **B.** Both qRT-PCR and immunoblotting demonstrated that TIMP-1 expression in normal human hepatocyte LO2 cells was significantly lower than that in 4 HCC cell lines (SK Hep1, Hep3B, HepG2 and Huh7).

Post-surgical follow-up information was obtained from 87 of the original 100 HCCs. The median time of follow-up was 25 months. The 87 HCC patients were divided into two groups: TIMP-1 high expression and TIMP-1 low/non expression using the median ratio of tumor/benign TIMP-1 expression as the cut-off value. The TIMP-1 high group included patients with higher TIMP-1 expression in HCC tissues, while the TIMP-1 low/non group included patients with lower or no TIMP-1 expression in tumor tissues. As shown in Table 2, most demographic and clinical characteristics were similar for the two groups, with the exception that there were more HCC patients with higher Edmonson–Steiner classification (*r* = 9.20, *P* = 0.002), advanced TNM stage (*r* = 9.10, *P* = 0.003), portal vein invasion (*r* = 13.86, *P* < 0.001) and intrahepatic metastases (*r* = 8.19, *P* = 0.004) in the TIMP-1 high group. We constructed Kaplan-Meier survival curves and found that the median overall survival was 23.46 months for HCC patients with elevated tumor tissue TIMP-1 expression (TIMP-1 high group), whereas the median overall survival was 58.17 months for HCC patients with lower TIMP-1 levels in adjacent liver tissues (TIMP-1 low/non group). The three-year survival rate was 41.8% for the TIMP-1 high group compared with 64.2% for the TIMP-1 low/non group. In a similar fashion, patients in the TIMP-1 high group (33.2%) had a reduced five-year survival rate compared with patients in the TIMP-1 low/non group (49.7%). Comparison of Kaplan Meier overall survival curves demonstrated notably longer post-surgical survival in the TIMP-1 low/non group (*HR* = 1.972; 95% CI: 1.111, 3.497; *P* = 0.020; Figure 2A). Moreover, univariate analysis demonstrated that intrahepatic metastases, higher Edmondson-Steiner classification, advanced TNM staging and higher TIMP-1 expression in HCC tissues were worse prognosis factors (Table 3). Multivariate Cox proportional-hazards regression analysis demonstrated that intrahepatic metastases, advanced TNM staging and higher TIMP-1 expression in HCC tissues were independent prognostic factors (Table 3). These data strongly support the idea that TIMP-1 is aberrantly up-regulated in HCC tissues, which predicts worse prognosis for patients with HCC after liver resection. The expression of TIMP-1 was detected in HCC cell lines including Huh7, Hep3B, HepG2 and SK Hep1 and the normal human hepatocyte cell line LO2 by RT-PCR and immunoblotting. Among these 5 cell lines, the lowest level of TIMP-1 expression was found in LO2 cells (Figure 2B).

**Table 2:** Demographic information and clinical features of 87 patients with follow-up information

Clinicopathological features		No. of patients		χ^2^	*p*
		TIMP-1 high	TIMP-1 low/non		
Age (year)	< 50	20	6	0.38	0.539
	≥ 50	43	18		
Gender	Male	53	25	3.49	0.062
	Female	19	3		
HBV infection	Present	56	19	1.382	0.240
	Absent	7	5		
Serum AFP level (ng/mL)	≥ 400	50	33	0.91	0.342
	< 400	13	5		
Tumor size (cm)	≥ 5	51	22	1.48	0.224
	< 5	12	2		
Liver cirrhosis	Present	61	23	0.05	0.821
	Absent	2	1		
Vasculature invasion	Present	26	10	< 0.01	0.973
	Absent	37	14		
Edmondson-Steiner Classification	I + II	14	13	9.20	0.002
	III + IV	49	10		
TNM stage	I + II	40	23	9.10	0.003
	III + IV	23	1		
Portal vein invasion	Present	5	10	13.86	< 0.001
	Absent	58	14		
Intrahepatic metastases	Present	4	7	8.19	0.004
	Absent	59	17		

**Table 3:** Cox-regression analysis of the relationship between the clinicopathological characteristics and overall survival rate of HCC patients after liver resection

Clinicopathological features	Univariate Analysis		Multivariate Analysis	
	RR (95% CI)	*p* Value	RR (95% CI)	*p* Value
Intrahepatic metastases	3.152 (1.569 – 5.682)	0.009	2.972 (1.457 – 4.632)	0.001
Higher Edmondson-Steiner classification	2.322 (1.252 – 3.210)	0.003	1.854 (0.899 – 2.152)	0.010
Advanced TNM staging	3.232 (2.012 – 4.365)	0.008	1.989 (1.228 – 3.788)	0.030
Higher TIMP-1 expression in HCC tissues	3.287 (1.622 – 5.751)	0.032	2.978 (1.457 – 5.125)	0.009

### Ectopic expression of TIMP-1 in Huh7 cells drives the transformation of LFs into CAFs

**Figure 3:**
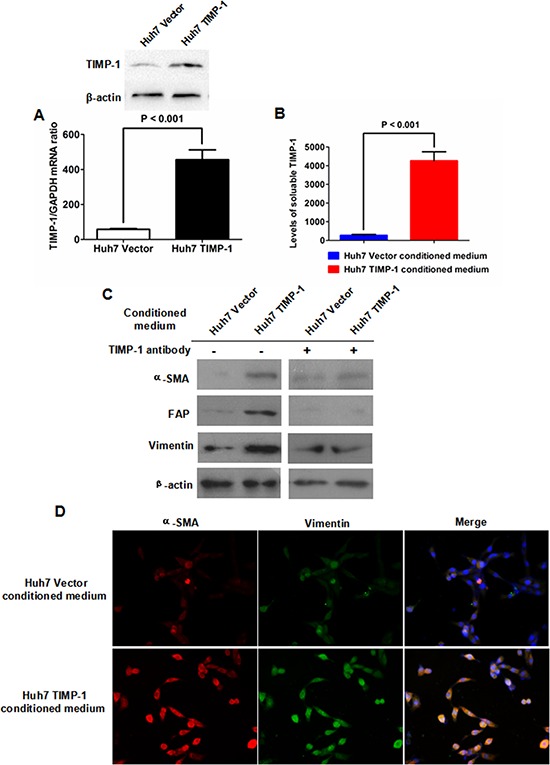
Enforced expression of TIMP-1 in Huh7 cells activated the transformation from LFs to CAFs **A.** Both qRT-PCR and immunoblotting demonstrated that Huh7 TIMP-1 cells express significantly more TIMP-1 than Huh7 Vector cells. **B.** An ELISA assay demonstrates that there was markedly higher soluble TIMP-1 in Huh7 TIMP-1-conditioned medium than in conditioned medium from Huh7 Vector cells (*P* < 0.001). **C.** Conditioned medium from Huh7 TIMP-1 cells increased the expression of α-SMA, FAP and vimentin in LFs compared with Huh7 Vector-conditioned medium. In addition, TIMP-1 antibody treatment abrogated the impact of Huh7 TIMP-1-conditioned medium on the expression of α-SMA, FAP and vimentin in LFs. **D.** Immunofluorescence staining confirmed that conditioned medium from Huh7 TIMP-1 cells lead to increased expression of α-SMA and vimentin in LFs compared with Huh7 Vector-conditioned medium.

Huh7 cells were transfected with a TIMP-1-expression plasmid or the pCMV-Tag2B vector followed by selection with G418 to yield stable TIMP-1-expressing Huh7 cells (Huh7 TIMP-1) and a stable control plasmid transfectant (Huh7 Vector). Both qRT-PCR and immunoblotting verified that TIMP-1 expression in Huh7 TIMP-1 cells was significantly higher than that in Huh7 Vector cells (Figure 3A). Both Huh7 TIMP-1- and Huh7 Vector-conditioned medium were harvested and used to culture LFs. ELISA measurements demonstrated that there was a remarkably higher level of TIMP-1 in conditioned medium from Huh7 TIMP-1 cells than Huh7 Vector cells (Figure 3B). As shown in Figure 3C, immunoblotting demonstrated that conditioned medium from Huh7 TIMP-1 cells resulted in significantly higher expression of α-SMA, FAP and vimentin in LFs compared with that in Huh7 Vector cells, which was abolished by the human TIMP-1 antibody. Immunofluorescence staining confirmed that there was higher expression of α-SMA and vimentin in LFs cultured with conditioned medium from Huh7 TIMP-1 cells as well (Figure 3D).

**Figure 4:**
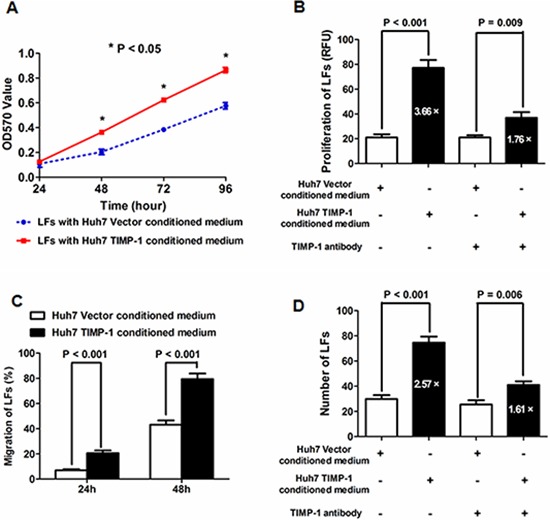
Conditioned medium from Huh7 TIMP-1 cells accelerated the cell viability, proliferation, migration and invasion of Huh7 cells **A.** The cell viability of LFs cultured with Huh7 TIMP-1-conditioned medium was significantly higher than LFs cultured with Huh7 Vector-conditioned medium at 48, 72 and 96 h as assessed by MTT assay. **B.** A BrdU incorporation assay showed that Huh7 TIMP-1-conditioned medium promoted cell proliferation in contrast with Huh7 Vector-conditioned medium; however, TIMP-1 antibody substantially repressed the up-regulation of LF proliferation induced by Huh7 TIMP-1-conditioned medium (increase of 3.66-fold *vs*. increase of 1.76-fold). **C.** The migration rate of LFs cultured with Huh7 TIMP-1 conditioned medium was apparently higher than those cultured with conditioned medium from Huh7 Vector cells at 24 and 48 hours after scratching as measured by scratching wound healing assay. **D.** There were more LFs crossing the Matrigel gel and filter of Transwell chambers in the Huh7 TIMP-1-conditioned medium group compared with the Huh7 Vector-conditioned medium group (*P* < 0.001). In addition, TIMP-1 antibody treatment suppressed the pro-invasion function of Huh7 TIMP-1-conditioned medium.

The results of MTT assays showed that the cell viability of LFs was significantly enhanced at 48, 72 and 96 h (all *P* values < 0.05, Figure 4A) by conditioned medium from Huh7 TIMP-1 cells compared with that from Huh7 Vector cells. We also quantitated cell proliferation using BrdU incorporation assays and confirmed that Huh7 TIMP-1 conditioned medium markedly accelerated the cell proliferation of LFs (*P* = 0.007, Figure 4B), which was abated by the human TIMP-1 antibody. Next, we conducted scratch wound healing assays and found that the migration rate of LFs cultured by Huh7 TIMP-1 conditioned medium was apparently higher than that with conditioned medium from Huh7 Vector cells at both 24 and 48 hours (*P* = 0.005 and *P* = 0.004, respectively, Figure 4C). As examined by Transwell invasion assays, it was found that the invasion ability of LFs with conditioned medium from Huh7 TIMP-1 cells was apparently higher (*P* < 0.001, Figure 4D) than LFs with Huh7 Vector conditioned medium, and human TIMP-1 antibody attenuated the function of Huh7 TIMP-1 conditioned medium. These data demonstrate that TIMP-1 is secreted by HCC cells and induces the transformation LFs into CAFs.

### CAFs driven by TIMP-1 facilitate the growth of HCC cells *in vitro* and *in vivo*

**Figure 5:**
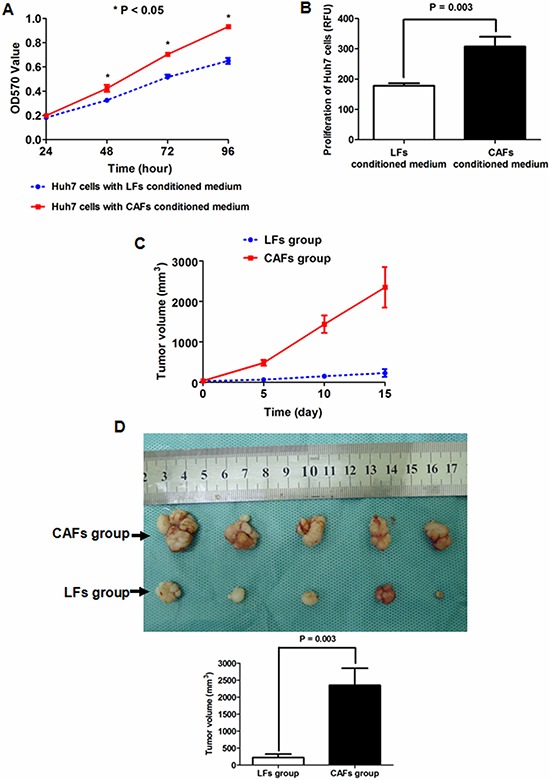
CAF-conditioned medium promoted the growth of HCC cells *in vitro* and *in vivo* **A.** An MTT assay showed that the cell viability of Huh7 cells was increased by CAF-conditioned medium compared with LF-conditioned medium at 48, 72 and 96 h. **B.** As assessed by BrdU incorporation assay, Huh7 cell proliferation was enhanced by CAF-conditioned medium compared with LF-conditioned medium (*P* = 0.003). **C.** The size of xenografts driven from CAFs and Huh7 cells (CAF group) was significantly greater than those driven by LFs and Huh7 cells (LF group) at 5, 10 and 15 days. **D.** At 15 days after cell injection, xenografts were obtained, and the Student's *t* test demonstrated that xenografts from the CAF group were significantly larger than those from the LF group (*P* = 0.003).

To evaluate the contribution of CAFs in terms of HCC cell growth, we cultured Huh7 cells with conditioned medium from LFs or CAFs. As evaluated by MTT assay, conditioned medium from CAFs inhibited the cell viability at 48, 72 and 96 h (all *P* values < 0.05, Figure 5A) compared with that from LFs. Consistent with cell viability, BrdU incorporation measurements also demonstrated that cell proliferation was apparently induced by conditioned medium from CAFs in contrast with medium from LFs (*P* < 0.001, Figure 5B), which indicates that TIMP-1 promotes HCC cell growth.

To further confirm the regulatory function of CAFs on HCC growth, we established a HCC xenograft system as described in the Methods section. Five nude mice were subcutaneously injected with a mixture of CAFs and Huh7 cells to for the CAF group. In addition, an LF group was established by subcutaneously injecting LFs and Huh7 cells in 5 other nude mice. Tumor size measurements taken every 3 days showed that the HCC xenografts in the CAF group grew faster than those in the LF group (Figure 5C). At 15 days after cell injection, HCC xenografts were harvested and measured by calipers. As shown in Figure 5D, co-injection with CAFs lead to the formation of larger subcutaneous HCC xenografts compared with co-injection with LFs. These data suggested that CAFs activated by TIMP-1 accelerated HCC growth *in vitro* and *in vivo*.

### CAFs induced by TIMP-1 repress HCC apoptosis through SDF-1/CXCR4/PI3K/AKT signaling

**Figure 6:**
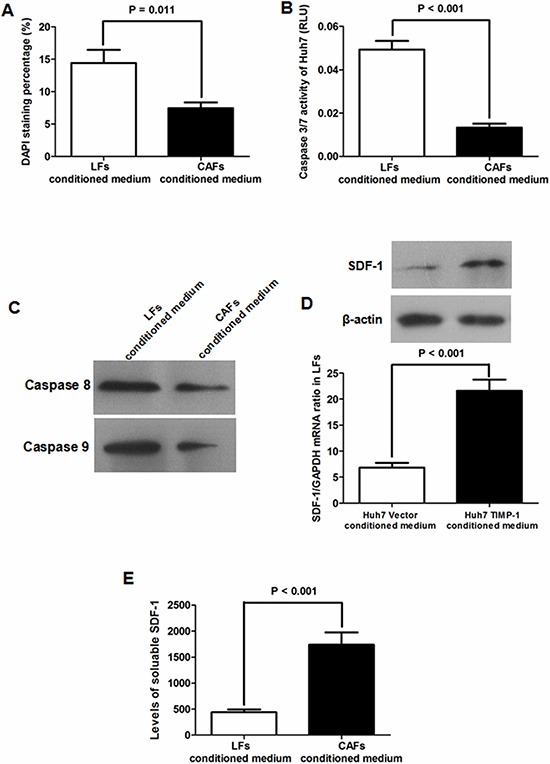
CAFs inhibited Huh7 apoptosis via activating SDF-1/CXCR4 pathway **A.** DAPI staining showed that there was a significantly higher apoptotic percentage in Huh7 cells grown with CAF-conditioned medium (CAF-conditioned medium group) compared with those cultured with LF-conditioned medium (LF-conditioned medium group) (*P* = 0.011). **B.** The caspase 3/7 activity of Huh7 cells in CAF-conditioned medium was significantly higher than that in LF-conditioned medium (*P* < 0.001). **C.** As measured by immunoblotting, there was apparently less expression of caspase 8 and caspase 9 in Huh7 cells cultured with CAF-conditioned medium. **D.** Both qRT-PCR and immunoblotting showed that there was more SDF-1 expression in LFs cultured with Huh7 TIMP-1-conditioned medium than LFs cultured with Huh7 Vector-conditioned medium. **E.** There was dramatically more soluble SDF-1 in conditioned medium from CAFs compared with LF-conditioned medium (*P* < 0.001) as assessed by ELISA assay.

To explore the mechanism by which CAFs activated by TIMP-1 promote HCC growth, we tested whether CAFs affected HCC apoptosis. DAPI staining assays showed that CAF-conditioned medium resulted in an approximately 50% decrease in Huh7 apoptosis compared with LF-conditioned medium (*P* = 0.011, Figure 6A). It was also found that conditioned medium from CAFs suppressed the activity of caspase 3/7, which was comparable to LF conditioned medium (*P* < 0.001, Figure 6B). In addition, immunoblotting assays revealed that CAF-conditioned medium lead to less expression of caspases 8 and 9 in Huh7 cells (Figure 6C), strongly suggesting that CAFs contribute to HCC growth by suppressing apoptosis.

**Figure 7:**
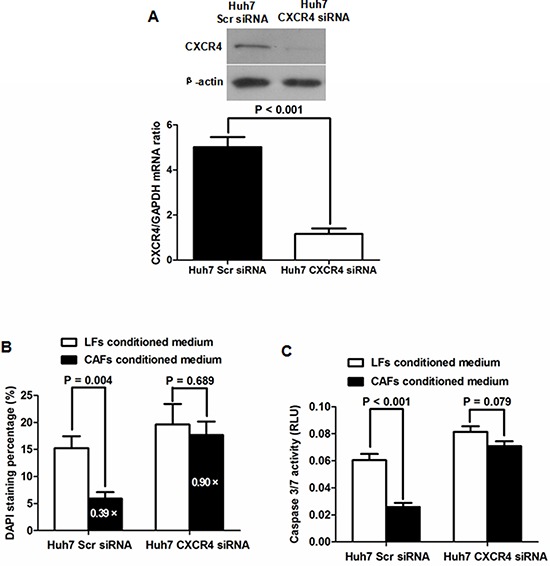
The SDF-1/CXCR4 axis is critical for maintaining the inhibitory effects of CAFs on HCC cells **A.** As examined by qRT-PCR and immunoblotting, siRNA sequences directed against CXCR4 successfully repressed the expression of CXCR4 in Huh7 cells. **B.** Knockdown of CXCR4 attenuated the anti-apoptotic function of CAF-conditioned medium in Huh7 cells (a reduction of 10% compared with a reduction of 61%). **C.** CAFs did not significantly affect caspase 3/7 activity without CXCR4 expression in Huh7 cells.

**Figure 8:**
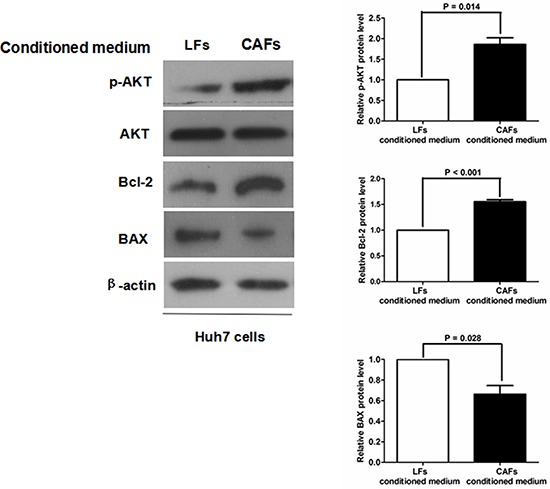
CAFs lead to greater AKT phosphorylation and an increased Bcl-2/BAX ratio in Huh7 cells Immunoblotting revealed that there was greater expression of phosphorylated AKT (p-AKT) and Bcl-2 and less BAX expression in Huh7 cells grown with conditioned medium from CAFs compared with LF-conditioned medium.

To clarify the underlying signaling pathway by which CAFs inhibit HCC apoptosis, we examined the expression level of SDF-1 protein, which has been found to be excreted by CAFs and is closely involved in cancer progression [16–18] in LFs with different conditioned medium by qRT-PCR and immunoblotting. As shown in Figure 6D, SDF-1 expression was significantly greater in LFs than in CAFs. Additionally, as presented in Figure 6E, the level of SDF-1 protein in CAF-conditioned medium was found to be approximately 3-fold higher than that in LF-conditioned medium as assessed by ELISA assay. It was logical to hypothesize that CAFs inhibit HCC apoptosis by activating the SDF-1/CXCR4 pathway. To test this hypothesis, we abated CXCR4 expression in Huh7 cells by siRNA and cultured Huh7 cells with either LF- or CAF-conditioned medium (Figure 7A). As shown in Figure 7B, the negative regulatory function of CAF-conditioned medium on Huh7 apoptosis nearly disappeared. Caspase 3/7 activity assessment also verified that knockdown of CXCR4 eliminated the antiapoptotic activity of CAF-conditioned medium on Huh7 cells (Figure 7C). These data demonstrate that the SDF-1/CXCR4 axis is essential for maintaining the inhibitory effects of CAFs on HCC cells. To further elucidate the underlying signal pathway, we examined downstream elements of the SDF-1/CXCR4 pathway in Huh7 cells cultured with either LF- or CAF-conditioned medium by immunoblotting. As shown in Figure 8, CAF-conditioned medium promoted the phosphorylation of AKT and increased the Bcl-2/BAX ratio.

**Figure 9:**
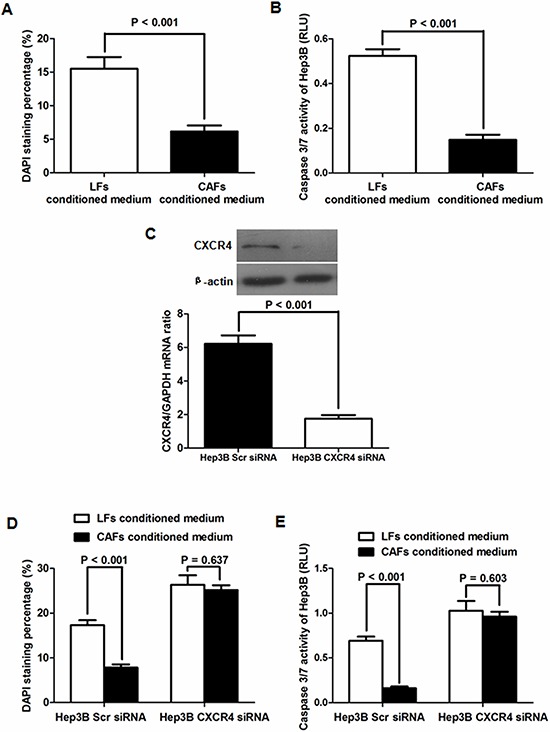
CAFs inhibit apoptosis by the SDF-1/CXCR4 pathway in Hep3B cells **A.** As assessed by DAPI staining, there was approximately 66.7% reduction in the apoptotic cell percentage in Hep3B cells cultured with conditioned medium from CAFs compared with LF-conditioned medium. **B.** Caspase 3/7 activity in Hep3B cells grown with CAF-conditioned medium was significantly lower than that in Hep3B cells grown with LF-conditioned medium. **C.** CXCR4 expression was successfully inhibited by siRNA, which was confirmed by qRT-PCR and immunoblotting. **D.** CAFs significantly decreased the percentage of apoptotic cells in Hep3B Scr siRNA cells, which had normal levels of CXCR4 expression. Conversely, in Hep3B CXCR4 siRNA cells in which CXCR4 was silenced, CAFs did not apparently impact the percentage of apoptotic cells. **E.** Consistent with the results of the DAPI staining assay, CAFs did not significantly influence the caspase 3/7 activity in Hep3B cells without CXCR4 expression.

Next, we grew Hep3B cells with either LF- or CAF-conditioned medium to confirm the findings between CAFs and Huh7 cells. Hep3B apoptosis was clearly inhibited by CAF-conditioned medium compared with LF-conditioned medium as assessed by DAPI staining (Figure 9A). There was a similar tendency found in caspase 3/7 activity assays (Figure 9B). We also silenced CXCR4 expression in Hep3B cells (Figure 9C) and found that there was no significant difference in the percentage of apoptotic Hep3B cells between the LF- and CAF-conditioned medium groups (Figure 9D). After repression of CXCR4 expression, CAF-conditioned medium did not apparently affect the caspase 3/7 activity (Figure 9E). These data indicate that CAFs initiated by TIMP-1 inhibit HCC apoptosis through SDF-1/CXCR4/PI3K/AKT signaling.

## DISCUSSION

HCC is the leading cause of cancer-related deaths worldwide. Surgical resection is the standard treatment for HCC; however, frequent tumor recurrence and/or metastasis results in the poor long-term outcome of HCC patients after liver resection. Therefore, it is urgent to explore the mechanism of HCC recurrence and metastasis after liver resection and identify efficacious predictive markers and therapy targets.

The expression of MMPs, which are closely related to cancer progression, is mediated at the transcriptional level by growth factors and transcriptional factors, and TIMPs modulate the activity of MMPS by proenzyme activation after secretion. Four various TIMPs have been reported, TIMP-1, TIMP-2, TIMP-3, and TIMP-4. Among these proteins, TIMP-1 has been the most extensively studied. TIMP-1 has been shown to abolish the activity of MMPs by forming 1:1 stoichiometric non-covalent complexes with endopeptidase and consequently plays a key role in maintaining the balance between ECM deposition and degradation in different physiological processes. Almost all members of the MMP family with the exception of MT-MMPs are inhibited by TIMP-1. As an endogenous inhibitor of MMPs, TIMP-1 was found to repress cancer cell growth and invasion by inhibiting the activity of MMPs [19–21]. However, in this study, we found that TIMP-1 is aberrantly up-regulated in 76.6% of HCCs, which are associated with worse prognosis. Intriguingly, our findings of TIMP-1 expression in HCC samples are consistent with results from Lempinen's group showing that HCC patients with low concentrations of serum TIMP-1 have significantly better overall survival than those with high concentrations of serum TIMP-1 [22]. Thus, TIMP-1 merely functions as a biomarker for HCC progression and contributes to accelerating cancer progression, which implies that it may serve as an important HCC therapeutic target.

To our knowledge, few studies have reported the mechanism by which TIMP-1 modulates HCC progression. A growing body of evidence has shown that CAFs, as a major component of the cancer stroma, fosters cancer cell growth and migration, and promotes tumor angiogenesis by secreting various growing factors and cytokines. Park and colleagues reported that activated CAFs exist in the HCC microenvironment [23]. In addition, a study from the group of Jia demonstrated that CAFs accelerate HCC growth *in vitro* and *in vivo* [24]. However, the underlying mechanism of how CAFs are initiated in the HCC microenvironment is largely unknown. In this study, we enhanced TIMP-1 expression in Huh7 cells with a TIMP-1 expression plasmid and found that the level of TIMP-1 in medium from Huh7 TIMP-1 cells was increased dramatically. Then, LFs driven from human normal liver were cultured with conditioned medium from either Huh7 TIMP-1 or Huh7 Vector cells, and we found that the viability, proliferation, migration and invasion of LFs were notably enhanced, and the expression of CAF markers including α-SMA, FAP and vimentin in LFs was significantly increased by conditioned medium from Huh7 TIMP-1 cells, which strongly supports that the TIMP-1 secreted by HCC cells in the tumor microenvironment initiates the transformation from LFs to CAFs. In a previous study, the increase in cell proliferation induced by CAFs (isolated from HCC tissues) and PTFs (isolated from adjacent liver tissues) was similar [24]. However, the *in vitro* and *in vivo* experiments in this study showed that CAFs accelerated HCC growth in a manner apparently comparable to LFs. It appears that CAFs initiated by different factors have diverse regulatory functions in HCC growth though a cluster of CAF markers is almost the same in these CAFs, indicating that there are complicated mechanisms through which CAFs mediate HCC progression. Furthermore, both DAPI staining and caspase 3/7 activity assays demonstrated that CAF conditioned medium remarkably represses HCC apoptosis. SDF-1 has been found to exert an inhibitory function on cancer apoptosis by binding with its primary receptor CXCR4, which is a seven transmembrane G-protein coupled receptor [25–27]. ELISA assays revealed that there was a dramatically higher level of soluble SDF-1 in CAF-conditioned medium than in LF-conditioned medium. Then, it was also found by immunoblotting that conditioned medium from CAFs enhanced AKT phosphorylation and increased the Bcl-2/BAX ratio. These data highly indicate that CAFs release significant amounts of soluble SDF-1 into the cancer microenvironment and activate SDF-1/CXCR4 signaling in neighboring HCC cells. Consequently, the PI3K/AKT pathway was trigged and then inhibited HCC apoptosis by up-regulating the Bcl-2/BAX ratio. In contrast, knockdown of CXCR4 in HCC cells abrogated the anti-apoptotic function of CAFs, which further confirmed that the SDF-1/CXCR4 pathway plays an essential role in the repression of cancer apoptosis mediated by CAFs in the HCC microenvironment.

**Figure 10:**
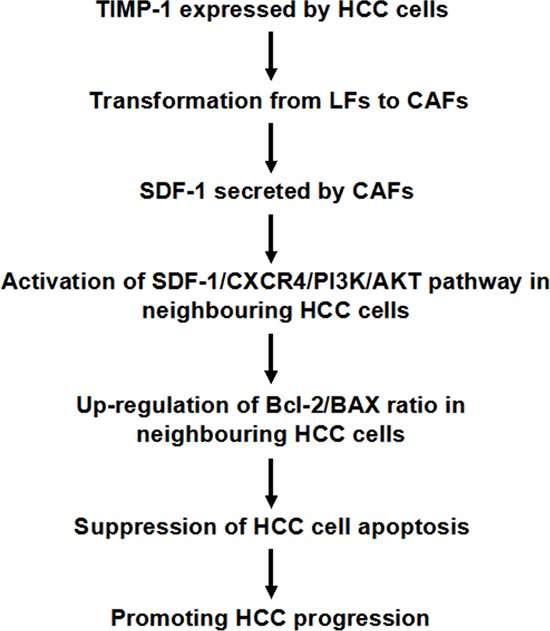
Working model of the mechanism by which TIMP-1 promotes HCC progression by initiating CAFs in the tumor microenvironment The data in this study reveal that aberrant overexpression of TIMP-1 in HCC cells initiate transformation from LFs to CAFs. Consequently, CAFs secreted soluble SDF-1 into the HCC microenvironment and activate SDF-1/CXCR4/PI3K/AKT signaling in neighboring HCC cells, which ultimately inhibit HCC apoptosis by up-regulating the Bcl-2/BAX ratio and accelerating HCC progression.

In summary, as presented in Figure 10, this study demonstrated that up-regulation of TIMP-1 in HCC cells results in transformation from LFs to CAFs. Then, CAFs secreted significant amounts of soluble SDF-1 into the HCC microenvironment and activated SDF-1/CXCR4/PI3K/AKT signaling in neighboring HCC cells, which ultimately inhibited HCC apoptosis by up-regulating the Bcl-2/BAX ratio. These findings have important implications for understanding the molecular mechanisms of CAF activation in the HCC environment but also for modulation of HCC by CAFs. Finally, these data indicate that TIMP-1 appears to be an efficacious predictive factor for HCC outcome after liver resection.

## MATERIALS AND METHODS

### Patients and tissues

There were a total of 100 HCC patients enrolled in the Department of Hepatobiliary Surgery at the First Affiliated Hospital of Xi'an Jiaotong University between January 2004 and June 2006. After examination by abdominal ultrasonography and computed tomography, all patients underwent a liver resection (curative resection for early HCC and palliative resection for advanced HCC). None of the patients received neo-adjuvant chemotherapy or radiotherapy before surgery. During liver resection, HCC and adjacent liver samples (> 2 cm distance from the margin of the resection) were collected and immediately stored in paraformaldehyde for IHC assays. Clinicopathological data were collected from medical records and summarized in Table 1. These clinicopathological features including Edmonson classification, clinical tumor-node-metastasis (TNM) grading and maximum tumor diameter, which were confirmed by an experienced pathologist. After surgery, all patients were followed up from 15 to 120 months, and follow-up information was obtained from 87 patients.

### Immunohistochemical staining

IHC staining assays were performed according to the protocol described previously [13]. Formalin-fixed and paraffin-embedded specimens were cut into 4 μm-thick sections, deparaffinized with dimethylbenzene and rehydrated in 100%, 95%, 90%, 80% and 75% ethanol. After washing in phosphate-buffered saline (PBS), specimens were boiled in antigen-retrieval buffer containing 0.01 M sodium citrate-hydrochloric acid (pH = 6.0) for 15 min. The slides was rinsed with PBS and blocked overnight at 4°C. After three washes in PBS, the slides were incubated with a mouse monoclonal antibody directed against TIMP-1 (Abcam, USA, at a 1:50 dilution) at 4°C overnight. Then, the primary antibody was incubated with biotinylated secondary antibody (Zhongshan Goldenbridge Biochnology). The bound antibody was visualized using HRP-streptavidin conjugates. The sections were counterstained with hematoxylin, dehydrated in alcohol and xylene, and finally mounted onto glass slides.

Slides were examined separately by two independent experienced pathologists blinded to the results of the other. The staining intensity was scored as four grades: 0 (negative), 1 (weakly positive), 2 (moderately positive), and 3 (strongly positive). The percentage of positive cells was also expressed in 5 categories where a score of 0 was given for 0–5%, 1 for 6–25%, 2 for 26–50%, 3 for 51–75%, and 4 for > 75%. The staining intensity and average percentage of positive cells were measured for 10 independent high magnification (400×) fields. The ultimate staining score was obtained by multiplying the staining intensity and percentage of positive cells. A third joint observation with conclusive agreement was conducted when discrepancies between investigators occurred.

### HCC cell lines and cell culture condition

Human HCC cell lines including Huh7, SK Hep1, Hep3B and HepG2, and the human immortalized normal hepatocyte cell line LO2 were purchased from the Institute of Biochemistry and Cell Biology, Chinese Academy of Sciences (Shanghai, China) and grown in DMEM medium with 10% fetal calf serum (FBS) in a 5% CO_2_ humidified atmosphere at 37°C.

### Primary LFs isolation and immortalization

Normal liver samples were obtained from hepatic hemangioma patients. After washing with PBS containing 100 U/ml penicillin and 100 μg/mL streptomycin, liver tissues were minced into small pieces and digested for 8 h at 37°C in DMEM supplemented with 10% FBS, 1 mg/ml collagenase type I and 100 U/mL hyaluronidase. Cell pellets were centrifuged and washed twice with PBS. Then, the cells were re-suspended in DMEM containing 10% FBS and cultured at 37°C in a humidified 5% CO_2_ environment. After 3 passages, the percentage of purified LFs reached approximately 95% as examined by immunofluorescence staining using antibodies targeting α-SMA, FAP and vimentin. LFs were immortalized by hTERT. The pBABEpuro-hTERT retroviral vector was transfected into PT67 cells with the FuGENE6 transfection reagent from Promega (Madison, WI). Retroviruses with hTERT were collected 48 h after transfection and used to infect LFs.

### Ethics statement

The protocols used in this study were reviewed and approved by Xi'an Jiaotong University Ethics Committee according to the Helsinki Declaration of 1975, and all patients recruited in this study have signed informed consent to participate in this study and for publication.

### Western immunoblotting

The immunoblotting assay was performed as described previously [14]. Briefly, cell lysates were prepared in RIPA buffer containing inhibitors for proteases and sodium orthovanadate as a inhibitor of phosphatases. After measuring the protein concentration using the BCA Protein Assay Kit from Thermo Scientific (Rockford, IL, USA), equal amounts (30 μg) of cell lysates were separated by electrophoresis in SDS-PAGE gels and transferred to polyvinylidene fluoride membranes. After probing overnight with primary antibodies including TIMP-1, AKT, phosph-AKT, α-SMA, FAP, vimentin, SDF-1, caspase 8 and caspase 9, blots were incubated with anti-mouse secondary antibodies conjugated with HRP (Abcam, USA), and signals were visualized using the HyGLO HRP detection kit from Denville (Metuchen, NJ, USA). β-actin was used to control for equal loading.

### TIMP-1 expressing plasmid and transfection

The cDNA of TIMP-1 was cloned into the pCMV-Tag2B vector from Stratagene (Santa Clara, CA). TIMP-1-negative Huh7 cells were transfected with the TIMP-1-expression plasmid using the FuGENE6 transfection reagent and designated Huh7 TIMP-1 expressing cells (Huh7 TIMP-1). The pCMV-Tag2B vector was transfected into Huh7 cells as Huh7 control cells (Huh7 Vector). After a two-week selection with geneticin (Invitrogen, Carlsbad, CA) at a dose of 500 μg/mL, we obtained stable transfection clones.

### Preparation of conditioned medium and enzyme-linked immunosorbent assay (ELISA)

Huh7 TIMP-1 cells were grown for 48 h to approximately 90% confluence. Then, these cells were cultured with 0.5% FBS DMEM for 36 h. After centrifugation, the supernatants was harvested, passed through a sterile Millipore filter with a 0.45 mm polyvinylidenedifluoride membrane to generated conditioned medium from Huh7 TIMP-1 cells. The conditioned medium from Huh7 Vector cells was obtained in the same manner. All conditioned medium were maintained in liquid nitrogen for later use.

To detect the level of soluble SDF-1 secreted by LFs or CAFs, 2 × 10^5^ LFs or CAFs were seeded into 6-well plates and grown for 48 h, and the supernatant was harvested for ELISA. The human CXCL12/SDF-1α quantikine ELISA kit from R&D Systems (Minneapolis, MN, USA) was used to perform ELISAs for SDF-1 measurement according to the manufacturer's instructions. In the same manner, we examined the level of soluble TIMP-1 in conditioned medium from Huh7 TIMP-1 and Huh7 Vector cells. There were 3 replicates for each measurement, and the assessment was repeated six times.

### Quantitative reverse-transcription-polymerase chain reaction (RT-PCR)

Total RNA for HCC cells as extracted using the RNeasy kit from Qiagen Co. (Valencia, CA), and cDNA was synthesized from 1 μg RNA with the PrimeScript RT Master Mix (TaKaRa, Osaka, Japan). RT-PCR analyses were performed using SYBR Premix Ex Taq (TaKaRa). The standard PCR conditions were 95°C for 30 sec followed by 40 cycles of 95°C for 5 sec and 60°C for 34 sec with a final dissociation stage, and the samples were run with an ABI 7300 system. GAPDH was simultaneously amplified in a separate set of tubes as control. The primer sequences used were as follows: TIMP-1: 5′-GGGGCTTCACCAAGACCTAC-3′ (forward) and 5′-GGAAGCCCTTTTCAGAGCCT-3′ (reverse); CXCR4: 5′-GATCAGCATCGACTCCTTCA-3′ (forward) and 5′-GGCTCCAAGGAAAGCATAGA-3′ (reverse); and GAPDH: 5′-ACCACAGTCCATGCCATCAC-3′ (forward) and 5′-TCCACCACCCTGTTGCTGTA-3′ (reverse). There were 6 replicates in each assessment, and each was repeated three times.

### RNAi transfections

siRNA sequences directed against CXCR4 (sc-35421) and scrambled siRNA sequences were from Santa Cruz Biotechnology (Santa Cruz, CA). HCC cells were seeded in six-well plates at 0.2 × 10^6^ per well and cultured until 70% cell confluence was reached. Cells in each well were transfected with 100 nM siRNA using Lipofectamine RNAi MAX Reagent from Invitrogen (Carlsbad, CA) according to the instruction manual.

### Cell viability assay

MTT (3-(4, 5-dimethylthiazol-2-yl)-2, 5-diphenyltetrazolium bromide) assays were used to measure cell viability. Briefly, cells were seeded in 96-well plates at 1 × 10^4^ cells/well and stained with 100 μl MTT (0.5 mg/ml) for 4 h at 37°C. Afterward, the culture medium was removed, and 150 μl dimethyl sulfoxide from Sigma-Aldrich (St. Louis, MO) was added in each well. The absorbance was assessed at 570 mm. All assessments were repeated at least six times.

### Cell proliferation assay

The level of cell proliferation was determined by estimating DNA uptake of 5-bromo-2′-deoxyuridine- 5′-monophosphate (BrdU). Cells were plated into 96-well plates at 5,000 cells per well for one day and measured using the BrdU ELISA kit from Roche (Indianapolis, IN) according to the instruction manual. All experiments were performed in sextuplicate.

### Apoptosis detection

Two distinct assessments were performed to measure apoptosis. First, 4′, 6-Diamidino-2-phenylindole (DAPI) staining assays were conducted. Briefly, cells were cultured to subconfluency on 6-well plates. After washing with PBS twice, the monolayer of cells was fixed with 1% paraformaldehyde for 10 min at room temperature. Then, fixed cells were incubated with 1 μg/ml DAPI for 5 min. Apoptosis was detected under fluorescence microscopy to determine apoptotic nuclear changes (chromatin condensation and nuclear fragmentation) after DAPI staining. Next, caspase 3/7 activity was examined for apoptosis detection. According to the manufacturer's instruction, cells were seeded in black 96-well plates at 1 × 10^4^ cells per well, grown for 24 h and then assessed using the Apo-ONE homogeneous Caspase-3/7 assay kit from Promega (Madison, WI, USA). Data were obtained using a Luminoskan. All experiments had 6 replicates were repeated 3 times.

### Migration and invasion assays

Scratch wound healing assays were used to determine the cell migration of LFs and CAFs. Confluent cells were grown in six-well culture plates and scraped from the bottom of culture plates using a pipette tip to create a cell-free area. The cell cultures were washed with PBS to remove cell debris. Cell migration was observed and photographed at 0 and 48 h post-scratch with microscope, and the percentage wound closure relative to that measured at 0 h was calculated.

For cell invasion assays, transwell inserts were pre-coated with Matrigel basement membrane matrix. Then, 2 × 10^5^ HCC cells were seeded on 8 μm with serum-free DMEM medium. DMEM medium with 20% FBS was added to the lower chamber as a chemoattractant. After 24 h, HCC cells that invaded through the Matrigel-coated transwell inserts were fixed and stained with 0.5% crystal violet in 20% methanol for 10 min. The number of invading cells was counted under microscopy.

### Immunofluorescence staining

As described previously [15], cells were plated on a cover glass in a 60-mm culture dish at 1 × 10^5^ cells/mL and incubated for 24 hours. After rinsing with Dulbecco's PBS (D-PBS), cells were fixed in ice-cold methanol for 15 mins. Then, cells were rinsed with D-PBS and incubated in blocking buffer (5% normal goat serum and 5% glycerol in D-PBS) for 1 h at 37°C. The cells were incubated with primary antibodies directed against α-SMA (rabbit anti-human) and vimentin (mouse anti-human) overnight at 4°C. After rinsing with D-PBS, the cells were incubated with Alexa-Fluor-555-conjugated donkey anti-rabbit and Alexa-Fluor-488-conjugated goat anti-mouse secondary antibodies from Invitrogen (Carlsbad, USA) for 2 h at room temperature. After washing with D-PBS and distilled water, cell nuclei were stained with DAPI. The images were viewed with a confocal microscope (Carl Zeiss, Oberkochen, Germany).

### *In vivo* experiments

An HCC xenograft model was generated to determine the role of CAFs induced by TIMP-1 on tumor growth. Four- to six-week-old male nude mice were obtained from the Animal Experiment Center of Xian Jiaotong University (Xian, China). After suspension in 150 μL Matrigel, CAFs were co-injected subcutaneously with Huh7 cells (the ratio of CAFs to Huh7 cells was 3:1, and the total cell number in each injection was 4 × 10^6^) in the flanks of 5 mice (CAFs group). Similarly, 4 × 10^6^ LFs and Huh7 cells at a ratio of 1:3 were subcutaneously co-inoculated in the flanks of 5 mice, generating the LF group. We measured the tumor sizes with calipers every 5 days. All mice were sacrificed on the 15th day after cell injection. The size of HCC xenografts was calculated using the following formula: volume = A × B^2^ × 0.52 (A, length; B, width; all measurements were in millimeters).

### Statistical analysis

All values were presented as the means and standard errors of the mean. Differences between groups were compared using the Mann–Whitney or Student's *t* test. Differences between Kaplan–Meier curves for HCCs with high and low TIMP-1 expression in HCC tissues compared with adjacent liver tissues were analyzed by the log-rank test. Statistical significance was set at *P* < 0.05. Multivariate analysis was performed using SPSS V17.0 software (SPSS Inc., Chicago, IL, USA), and PRISM 5 (GraphPad, La Jolla, CA, USA) was used for other statistical analyses.

## Abbreviations

TIMP-1: tissue inhibitor of metalloproteinases 1
HCC: hepatocellular carcinoma
MMPs: matrix metalloproteinases
IHC: immunohistochemistry
siRNA: small interfering RNA
ECM: extracellular matrix
TNM: clinical tumor-node-metastasis
PBS: phosphate-buffered saline
MTT: 3-(4, 5-dimethylthiazol-2-yl)-2, 5-diphenyltetrazolium bromide
BrdU: 5-bromo-2′-deoxyuridine-5′-monophosphate
FBS: fetal calf serum
TNM: Tumor Node Metastasis
AFP: serumα-fetoprotein

## References

[1] Bosch FX, Ribes J, Diaz M, Cleries R (2004). Primary liver cancer: worldwide incidence and trends. Gastroenterology.

[2] Buchheit CL, Weigel KJ, Schafer ZT (2014). Cancer cell survival during detachment from the ECM: multiple barriers to tumour progression. Nat Rev Cancer.

[3] Kostourou V, Papalazarou V (2014). Non-collagenous ECM proteins in blood vessel morphogenesis and cancer. Biochim Biophys Acta.

[4] Alcantara MB, Dass CR (2013). Regulation of MT1-MMP and MMP-2 by the serpin PEDF: a promising new target for metastatic cancer. Cell Physiol Biochem.

[5] Moore CS, Crocker SJ (2012). An alternate perspective on the roles of TIMPs and MMPs in pathology. Am J Pathol.

[6] Chirco R, Liu XW, Jung KK, Kim HR (2006). Novel functions of TIMPs in cell signaling. Cancer Metastasis Rev.

[7] Batra J, Robinson J, Soares AS, Fields AP, Radisky DC, Radisky ES (2012). Matrix metalloproteinase-10 (MMP-10) interaction with tissue inhibitors of metalloproteinases TIMP-1 and TIMP-2: binding studies and crystal structure. J Biol Chem.

[8] Roy R, Zurakowski D, Wischhusen J, Frauenhoffer C, Hooshmand S, Kulke M, Moses MA (2014). Urinary TIMP-1 and MMP-2 levels detect the presence of pancreatic malignancies. Br J Cancer.

[9] Bodnar M, Szylberg L, Kazmierczak W, Marszalek A (2014). Tumor progression driven by pathways activating matrix metalloproteinases and their inhibitors. J Oral Pathol Med.

[10] Cui H, Seubert B, Stahl E, Dietz H, Reuning U, Moreno-Leon L, Ilie M, Hofman P, Nagase H, Mari B, Kruger A (2014). Tissue inhibitor of metalloproteinases-1 induces a pro-tumourigenic increase of miR-210 in lung adenocarcinoma cells and their exosomes. Oncogene.

[11] Lokeshwar BL, Selzer MG, Block NL, Gunja-Smith Z (1993). Secretion of matrix metalloproteinases and their inhibitors (tissue inhibitor of metalloproteinases) by human prostate in explant cultures: reduced tissue inhibitor of metalloproteinase secretion by malignant tissues. Cancer Res.

[12] Mazzocca A, Dituri F, Lupo L, Quaranta M, Antonaci S, Giannelli G (2011). Tumor-secreted lysophostatidic acid accelerates hepatocellular carcinoma progression by promoting differentiation of peritumoral fibroblasts in myofibroblasts. Hepatology.

[13] Zheng X, Yao Y, Xu Q, Tu K, Liu Q (2010). Evaluation of glioma-associated oncogene 1 expression and its correlation with the expression of sonic hedgehog, E-cadherin and S100a4 in human hepatocellular carcinoma. Mol Med Rep.

[14] Gai X, Tu K, Lu Z, Zheng X (2014). MRC2 expression correlates with TGFbeta1 and survival in hepatocellular carcinoma. Int J Mol Sci.

[15] Zheng X, Vittar NB, Gai X, Fernandez-Barrena MG, Moser CD, Hu C, Almada LL, McCleary-Wheeler AL, Elsawa SF, Vrabel AM, Shire AM, Comba A, Thorgeirsson SS, Kim Y, Liu Q, Fernandez-Zapico ME (2012). The transcription factor GLI1 mediates TGFbeta1 driven EMT in hepatocellular carcinoma via a SNAI1-dependent mechanism. PLoS One.

[16] Dillenburg-Pilla P, Patel V, Mikelis CM, Zarate-Blades CR, Doci CL, Amornphimoltham P, Wang Z, Martin D, Leelahavanichkul K, Dorsam RT, Masedunskas A, Weigert R, Molinolo AA, Gutkind JS (2014). SDF-1/CXCL12 induces directional cell migration and spontaneous metastasis via a CXCR4/Galphai/mTORC1 axis. FASEB J.

[17] Bhardwaj A, Srivastava SK, Singh S, Arora S, Tyagi N, Andrews J, McClellan S, Carter JE, Singh AP (2014). CXCL12/CXCR4 signaling counteracts docetaxel-induced microtubule stabilization via p21-activated kinase 4-dependent activation of LIM domain kinase 1. Oncotarget.

[18] Ho IA, Yulyana Y, Sia KC, Newman JP, Guo CM, Hui KM, Lam PY (2014). Matrix metalloproteinase-1-mediated mesenchymal stem cell tumor tropism is dependent on crosstalk with stromal derived growth factor 1/C-X-C chemokine receptor 4 axis. FASEB J.

[19] Kawamata H, Kawai K, Kameyama S, Johnson MD, Stetler-Stevenson WG, Oyasu R (1995). Over-expression of tissue inhibitor of matrix metalloproteinases (TIMP1 and TIMP2) suppresses extravasation of pulmonary metastasis of a rat bladder carcinoma. Int J Cancer.

[20] Chen Y, Wei X, Guo C, Jin H, Han Z, Han Y, Qiao T, Wu K, Fan D (2011). Runx3 suppresses gastric cancer metastasis through inactivation of MMP9 by upregulation of TIMP-1. Int J Cancer.

[21] Gong Y, Scott E, Lu R, Xu Y, Oh WK, Yu Q (2013). TIMP-1 promotes accumulation of cancer associated fibroblasts and cancer progression. PLoS One.

[22] Lempinen M, Lyytinen I, Nordin A, Tervahartiala T, Makisalo H, Sorsa T, Isoniemi H (2013). Prognostic value of serum MMP-8, -9 and TIMP-1 in patients with hepatocellular carcinoma. Ann Med.

[23] Kim GJ, Rhee H, Yoo JE, Ko JE, Lee JS, Kim H, Choi JS, Park YN (2014). Increased expression of CCN2, epithelial membrane antigen, and fibroblast activation protein in hepatocellular carcinoma with fibrous stroma showing aggressive behavior. PLoS One.

[24] Jia CC, Wang TT, Liu W, Fu BS, Hua X, Wang GY, Li TJ, Li X, Wu XY, Tai Y, Zhou J, Chen GH, Zhang Q (2013). Cancer-associated fibroblasts from hepatocellular carcinoma promote malignant cell proliferation by HGF secretion. PLoS One.

[25] Pance A, Morrissey-Wettey FR, Craig H, Downing A, Talbot R, Jackson AP (2014). SDF-1 chemokine signalling modulates the apoptotic responses to iron deprivation of clathrin-depleted DT40 cells. PLoS One.

[26] Kuhne MR, Mulvey T, Belanger B, Chen S, Pan C, Chong C, Cao F, Niekro W, Kempe T, Henning KA, Cohen LJ, Korman AJ, Cardarelli PM (2013). BMS-936564/MDX-1338: a fully human anti-CXCR4 antibody induces apoptosis *in vitro* and shows antitumor activity *in vivo* in hematologic malignancies. Clin Cancer Res.

[27] Righi E, Kashiwagi S, Yuan J, Santosuosso M, Leblanc P, Ingraham R, Forbes B, Edelblute B, Collette B, Xing D, Kowalski M, Mingari MC, Vianello F, Birrer M, Orsulic S, Dranoff G (2011). CXCL12/CXCR4 blockade induces multimodal antitumor effects that prolong survival in an immunocompetent mouse model of ovarian cancer. Cancer Res.

